# A Systematic Review on Social Cognition in ADHD: The Role of Language, Theory of Mind, and Executive Functions

**DOI:** 10.3390/brainsci14111117

**Published:** 2024-11-01

**Authors:** Alessandra Capuozzo, Salvatore Rizzato, Giuseppe Grossi, Francesca Strappini

**Affiliations:** 1 Italian Society of Behavioral and Cognitive Therapy, SITCC, Viale Antonio Gramsci, 13, 80122 Naples, Italy; 2 Dipartimento Salute Mentale, Asl Roma 4, Via Trento, 20, 62, 00062 Bracciano, Italy; salvatore.rizzato@aslroma4.it; 3 Center for Psychotherapy and Rehabilitation “InMovimento”, Via Andrea Doria, 19-21, 04022 Fondi, Italy; g.grossi@hotmail.it; 4 Association School of Cognitive Psychology (APC-SPC), Viale Castro Pretorio 116, 00185 Rome, Italy; 5 Dipartiment of Systems Medicine, University of Rome “Tor Vergata”, Via Montpellier 1, 00133 Rome, Italy

**Keywords:** attention deficit hyperactivity disorder, social cognition, executive function, language, ToM

## Abstract

**Background:** In this systematic review, conducted according to the PRISMA 2020 guidelines, we aimed to assess differences in the cognitive processes associated with social cognition—namely language, theory of mind (ToM), and executive functions (EFs)—between ADHD and control groups. **Methods:** The review included studies indexed in PubMed, Google Scholar, and PsycINFO up until May 2024. Eligible original peer-reviewed articles met the following criteria: they were written in English, included a clinical group with a current primary ADHD diagnosis, were empirical, included quantitative data, and utilized standardized and validated measures with adequate psychometric properties to assess social cognitive processes. **Results and Discussion:** A total of 1215 individuals with ADHD participated in the selected studies. Out of the 22 articles reviewed, 17 reported significant differences between ADHD and the controls across several cognitive processes related to language and EF rather than ToM. These processes included pragmatic skills, verbal and nonverbal communication, emotional prosody, interaction skills, sarcasm, paradoxical sarcasm recognition, ambiguous situations, emotion recognition, false belief, social problem solving, social behaviors, and gesture codification. We also discuss the limitations of the research and the implications of our findings. Systematic review registration: PROSPERO ID: CRD42023474681.

## 1. Introduction

Social cognition comprises a broad range of processes underlying individuals’ capability to perceive, categorize, interpret, and predict others’ behavior and mental states [1]. Thus, it is one of the key abilities of humans to adapt to society and to feel connected to each other [2]. However, this ability may function differently in individuals with atypical development, such as attention deficit hyperactivity disorder (ADHD) [3,4]. According to the DSM-5 [5], three subtypes of ADHD can be identified: inattentive, hyperactive, and combined. Behavioral distinctions can be broadly categorized into externalized and internalized behaviors [6,7]. In the impulsive ADHD presentation, behaviors may include difficulty remaining quiet, challenges in engaging in sedentary activities, feelings of being under constant “pressure” due to an internal drive, and excessive talking. In contrast, the inattentive ADHD presentation is characterized by frequent oversight, difficulties in organizing tasks and managing time, distractibility from external stimuli, and hesitation in engaging in activities that require sustained mental effort. Finally, the combined ADHD presentation encompasses behaviors associated with both inattentive and hyperactive presentations. Although the principal manifestations of ADHD are impulsivity, hyperactivity, and inattention, most individuals with ADHD experience problems in relationships. Indeed, studies indicate high comorbidity between ADHD and personality disorders, estimated at around 62% [8,9], and in these specific disorders, the predominant symptom is difficulty in social relationships [5]. Apart from these conditions, individuals with ADHD generally experience social difficulties [10], suggesting atypical functioning in social abilities [11,12], such as abnormality in morality development [13] and play [14].

An explanation for these relationship problems could be consequences of ADHD manifestations, such as impulsivity, emotion dysregulation, need for novelty, and attention difficulties [15,16,17]. Indeed, impulsivity in social relations could result in broken friendships by saying or doing regrettable things or finding oneself in unpleasant situations. Emotion dysregulation worsens the process of monitoring and regulating emotions during social situations, leading to being overwhelmed with explosive emotions like anger. The need for novelty is significant as individuals with ADHD report boredom when meeting the same people or being in similar situations, leading them to isolate themselves from others for long periods. Moreover, attention difficulties result in not listening and neglecting necessary care for others [18].

Recent studies have highlighted another important point: the possibility that difficulties in social cognition are also due to aspects not closely linked to principal ADHD manifestations but rather to processes related to executive functions (EFs), language, and theory of mind (ToM) [19,20]. Indeed, when considering social cognition, it should be noted that it is a multidimensional function encompassing several processes, such as social decision making, understanding others’ mental states, and nonverbal communication. These cognitive processes are associated with different brain areas [2,19,21] and might function differently in ADHD compared to non-ADHD individuals. These processes have been traditionally clustered into three domains associated with (a) perceptual processing of social information such as emotional expressions (social perception), (b) understanding others’ affective or cognitive mental states (social understanding), and (c) planning behaviors, taking into consideration other agents’ choices in addition to their own goals (social decision making) [1]. In general, the most studied social cognitive processes include social perception, social knowledge, attributional bias, emotional processing [21], empathy, humor processing, gesture, language ability, and emotional expression recognition [19]. Emotional processing is the ability to feel emotions and use them during everyday life [22]; attributional bias is the ability to make inferences on causes of events, both positive or negative [21]; social knowledge is the awareness of unwritten social rules and the understanding of the importance of those [23]; social perception is the ability to recognize social rules and social contexts, such as understanding when it is appropriate to behave considering hierarchy in professional situations or deciding when a specific topic is suitable in a particular context [24]. However, in order to utilize each of these abilities, it is necessary to monitor internal and external stimuli, interpret them, and understand the best behavior considering the context and consequences [19]. Therefore, most of these steps require EFs, and it is well known that EFs work atypically in ADHD [25]. EFs are associated with a large-scale network of brain areas encompassing the frontal cortex, such as the inferior frontal gyrus, dorsolateral prefrontal cortex, rostral prefrontal cortex, orbitofrontal prefrontal cortex, anterior insula, and basal ganglia. These brain areas are associated with fundamental EF processes, such as shifting, working memory, inhibition, and making decisions [26]. These processes are necessary during social interaction, for instance, when it is requested to inhibit a specific behavior, listen to someone during confusing events, or decide what answer to implement in a specific situation.

Adapting to social contexts and empathizing with others also requires components such as reading verbal and nonverbal communication to understand the feelings and intentions of others, being aware of the feelings and intentions of the self, evaluating what is more appropriate considering the social situation, and then deciding how to communicate with others [19]. Thus, nonverbal communication abilities, such as pragmatics and prosody, continuously used during social interactions, are necessary components of social cognition. Pragmatics is the ability to use speech appropriately, like initiating and concluding conversations, comprehending the general context of a conversation, and maintaining coherence in storytelling [27]. Prosody is an essential part of phonology and entails a variety of phenomena, such as the study of the rhythm, stress, intonation, and phrasing of speech and how these features contribute to meaning [28]. Overall, prosody, pragmatics, and EFs are indispensable for maintaining social interactions, maintaining continuity in relationships, and following social rules. Several studies have indicated that social cognition disabilities are due to EFs [19,29,30] and language atypicality [29,31,32].

Another crucial aspect of social cognition is ToM. ToM refers to the capacity to comprehend the mental states and intentions of others. Abilities belonging to ToM include understanding desires, emotional responses, humor, and hidden intentions, which are necessary for social cognition to work correctly. However, it appears that ADHD, particularly in children and adolescents, exhibits poorer performance in ToM compared to typical development [33].

Despite indications that social cognition, coupled with language, EFs, and ToM might play a significant role in ADHD functioning, the literature accumulated so far has produced mixed results. Indeed, some studies have found no differences in social cognition in ADHD compared to non-ADHD individuals (e.g., [11,34,35,36]), while others showed significant results (e.g., [12,33,37]). Therefore, these discrepancies create a substantial gap in understanding ADHD functioning, and the effective role of the specific social cognitive processes remains unclear, with important implications in clinical practice. Thus, the present systematic review aimed to synthesize the available empirical studies assessing the social cognitive processes associated with EFs, language, and ToM (such as emotion recognition, empathy, and pragmatics) in relation to ADHD.

## 2. Materials and Methods

**Figure 1:**
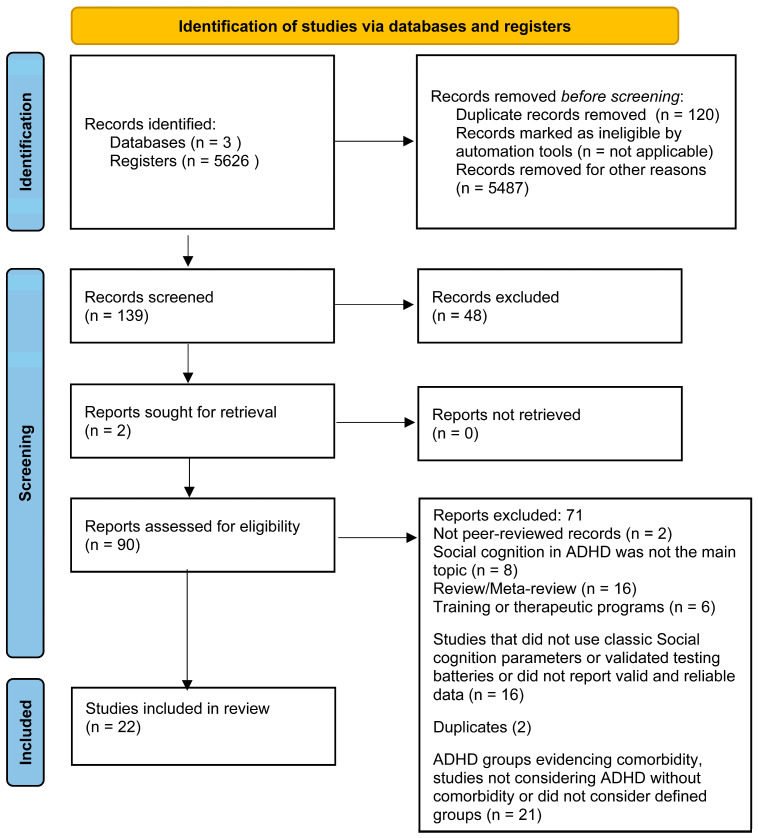
Diagram of the selection and filtering processes, according to the PRISMA checklist (Moher et al., 2009 [38]).

This systematic review was conducted following guidelines of the Preferred Reporting Items for Systematic Reviews and Meta-Analyses Guidelines (PRISMA 2020) [38,39] and preregistered in the International Prospective Register of Systematic Reviews (PROSPERO, CRD42023474681). The PRISMA protocol consists of a 27-item checklist (Supplementary Material, PRISMA 2020 checklist) and a 4-phase flow diagram that guides the systematic review process (see flow diagram in Figure 1 and checklist in Figure S1).

### 2.1. Research Strategies

The research was conducted on Google Scholar, Pubmed, and PsynINFO; no limit was entered for the publication years. The search was performed up to May 2024. Following the definition of social cognition used in other studies [19,21], we used the following Boolean combination of keywords: “ADHD” OR “Adult ADHD” OR “Attention Deficit Hyperactivity Disorder” AND “empathy” OR “social skills” OR “attributional bias” OR “emotional processing” OR “ToM” OR “theory of mind” OR “emotional cognition” OR “social cognition” AND “language” OR “executive functions” OR “working memory”.

We based the research question and strategy of our study on the Population, Intervention, Comparison, and Outcome (PICO) model, frequently used in evidence-based practice and recommended for systematic reviews [40]. Hence, the Population was individuals with ADHD; the Instrument was psychometric questionnaires measuring social cognition processes related to executive functions, theory of mind, and language; the Control was the group of non-ADHD individuals; and the Outcome was differences in social cognitive processes, as measured by the psychometric questionnaires between ADHD and non-ADHD individuals. Therefore, the final PICO question was “Are social processes related to executive functions, theory of mind, and language, as measured by psychometric questionnaires, different between ADHD and non-ADHD individuals?

We searched for additional articles in the reference lists of the selected papers (i.e., backward research) and identified studies that cited the selected articles (i.e., forward research). First, we performed the screening by reading the titles and abstracts. Then, we read the full texts of the selected studies. The articles that met the eligibility criteria based on the above screening are summarized in Table 1, Table 2 and Table 3.

### 2.2. Eligibility Criteria

Consistent with our aims (i.e., studying the association between ADHD and cognitive processes associated with social cognition), we included articles that fulfilled the following criteria: (a) original, peer-reviewed articles; (b) written in English; (c) included a clinical group with current primary ADHD diagnosis (DSM-defined ADHD or ICD-defined hyperkinetic disorder); (d) were empirical and included quantitative data (i.e., reviews, case studies, and qualitative papers); and (e) used a standardized and validated scale with adequate psychometric properties, such as reliability and validity, as core measures of social cognitive processes. Reliability assesses if random error is minimal and if an instrument produces stable results. Validity assesses if an instrument truly measures what it intends to study. The presence of these properties represents the underlying assumption of using patient-reported outcome assessment instruments. We excluded studies that presented (a) treatment or training programs and (b) included individuals with other comorbidities, such as autism spectrum disorder, learning disorders, tic disorder, anxiety disorders, and conduct disorders. We focused only on “pure” cases vs. comorbid cases to disentangle the association between social cognition processes and ADHD. For instance, EF problems may be associated with both autistic traits and ADHD [41], so including these comorbid cases may hide the ADHD-specific social cognition problems associated with EF.

Articles from all publication years were accepted to allow for a thorough and complete review.

We considered ADHD in each stage of life, including during childhood, adolescence, and adulthood.

### 2.3. Data Collection

Two authors (A.C. and S.R.) screened articles independently, first by title and abstract, then by full text, to determine eligibility for final inclusion. Data were extracted by the two authors independently, based on exclusion and inclusion criteria. Data extraction was limited to published data. We developed a data extraction sheet using Microsoft Excel (Microsoft 365 software), inserting relevant information for this review and checking the requirements for each study. At each screening stage, any differences between authors were discussed, and a consensus decision for eligibility and inclusion was made for all articles until inter-rater reliability of ≥90% of agreement was obtained. During data collection, descriptive and quantitative data were collected from each study and included in Table 1, Table 2 and Table 3. The information included (a) metadata (authors and year of publication), (b) type of group (clinical and control), (c) information related to the sample (sample size, gender, and age), (e) methodological information (i.e., the tests used to measure social cognition), (f) the cognitive functions and processes measured in each test, and (f) main results.

We reported data that investigated differences between ADHD and non-ADHD; this included Healthy Control (HC), Autism Spectrum Disorder (ASD), and Specific Learning Disorder (SLD) and comparisons between ADHD and non-ADHD and ADHD with comorbidity, e.g., Conduct Problems (CPs). The test considered acceptable to make ADHD diagnosis, according to DSM 5 and ICD criteria, was a cohort of tests that evaluate cognitive abilities (e.g., WISC-IV or WAIS), ADHD interview or semistructured interview administered to adult and child participants or children’s parents/teachers (e.g., BAARS and BRIEF), and diagnosis performed in public health facilities.

To extract data, we considered only validated tests, each focusing on a specific aspect of social cognition, considering the definition used in previous reviews [19,21]; this choice implied the selection of a wide range of tests. Given the multidimensionality of social cognition, for each study, we categorized the results of each test into three domains: (a) language, (b) ToM, and (c) EFs. Importantly, we did not report data that were not related to these three functions. We used Table 1, Table 2 and Table 3 to tabulate collected data divided into three macro groups.

We used the Joanna Briggs Institute (JBI) checklist to assess the quality of individual studies and the risk of bias [42]. This checklist is widely used in systematic reviews and meta-analyses to evaluate the reliability and validity of studies that investigate the presence of a condition at one single timepoint [42]. Two reviewers (A.C. and S.R.) independently and blindly assessed the quality until an agreement was reached. This checklist includes eight questions: (1) ‘Were the criteria for inclusion in the sample clearly defined?’; (2) ‘Were the study subjects and the setting described in detail?’; (3) ‘Was the exposure measured in a valid and reliable way?’; (4) ‘Were objective, standard criteria used for measurement of the condition?’; (5) ‘Were confounding factors identified?’; (6) ‘Were strategies to deal with confounding factors stated?’; (7) ‘Were the outcomes measured in a valid and reliable way?’; and (8) ‘Was appropriate statistical analysis conducted?’ [42]. Specifically, reviewers responded to these eight questions with ‘Yes’, ‘No’, ‘Unclear’, or ‘Not applicable’. A ‘Yes’ response contributed one point, while other responses did not contribute points. The total score, ranging from 0 to 8, was the sum of all ‘Yes’ responses. The overall quality assessment score was calculated by dividing the total score by the maximum possible score, expressed as a percentage. Studies were classified as low risk of bias if the total score was above 70%, moderate risk of bias between 50.0% and 70.0%, and high risk of bias if it was up to 49%.

Upon assessing the methodological quality, all studies were found to be of high quality, according to the total quality assessment score. The results of the evaluation of the included studies, conducted using the JBI checklist, are presented in the Supplementary Information (Table S1).

Due to the heterogeneity of the groups’ data, a meta-analysis was not possible.

## 3. Results

Figure 1 shows a diagram of the selection and filtering processes according to the PRISMA 2020 checklist. A total of 139 articles were selected based on their titles and abstracts. Out of the 139 records identified, 48 were excluded during the screening phase, and an additional 66 were excluded during the eligibility phase based on the inclusion criteria outlined in the methods section. In the identification phase, 137 articles were found, and 2 were identified through other papers. Table 1, Table 2 and Table 3 show the results of the final 22 articles selected. Among these, twelve studies examined social cognition in children (aged 7–12 years) [4,12,29,33,34,43,44,45,46,47,48,49], one in adolescence (aged 14–15 years) [50], and nine in adulthood (aged 20–40 years) [35,36,51,52,53,54,55,56,57].

Overall, 17 out of the 22 studies, focusing on the relationship between ADHD and social cognition, revealed significant results within three macro-cognitive functions: EFs [4,11,33,34,35,43,47,49,50,51,52,54,55,56,57], ToM [12,34,35,36,48,50,51,53,54,55,56], and language [11,12,29,33,43,44,45,46,47,48,50]. Conversely, six studies did not find any social cognition deficits associated with ADHD [34,35,36,52,53,54], with one study focusing on social cognition in childhood [34] and the rest in adulthood [35,36,52,53,54].

### 3.1. Language

In the included studies, several tests were used to evaluate social cognition skills requiring language. These tests assess social cognition through pragmatic skills, language understanding, appropriate verbal and nonverbal communication.

Table 1 describes the results associated with social cognition, considering processes that require language, that is, pragmatic skills, verbal and nonverbal communication, emotional prosody, interaction skills, sincerity, sarcasm, and paradoxical sarcasm recognition [11,12,29,43,44,45,46,47,48,50]. The results showed that several language processes seem to be impaired in children with ADHD. However, Cadesky et al. [43] did not find any difference in nonverbal communication when testing children divided into four groups: ADHD, ADHD and CP, CP, and neurotypical, using the DANVA paralanguage test. Conversely, in adults it was found that only emotional prosody, which was assessed with TAB, seemed to be impaired [11].

### 3.2. Theory of Mind

In the selected studies, ToM was investigated with several tests that assessed social cognition by considering emotional comprehension and the intentions of others. For instance, the Movie for the Assessment of Social Cognition (MASC) [58], used by Mehren et al. (2021) [36] and Abdel-Hamid et al. (2019) [55], was designed to evaluate social cognition through visual (facial emotion recognition), auditory, and verbal (language content) channels.

Table 2 describes the results of social cognition considering processes requiring ToM. The results show that the investigated processes included empathy [11], facial expression comprehension and social emotions [34,48,56], ToM knowledge [33], facial emotional expression comprehension [50], and emotional understanding [12,36,51,54,55].

Unlike the studies that assessed language, the results for ToM were contrasting. Indeed, Parke et al. (2021) [12], Çiray et al. [50], and Sahin et al. [48] found impairment in facial emotion expression comprehension in children with ADHD and Tatar and Cansiz [56] found the same result in adults. Conversely, Gonzalez-Gadea et al. [54], Abdel-Hamid et al. [55], Mehren et al. (2021) [36], Pitzianti et al. [34], Friedman et al. [51], and Ilzarbe et al. [53] did not find any impairment in emotional comprehension or ToM. Interestingly, in these studies, participants were adolescents or adults, suggesting that ADHD children may develop cognitive processes related to ToM later than non-ADHD individuals or learn it during adolescence and adulthood.

### 3.3. Executive Functions

Based on the measured processes, the test used to assess EFs can be classified into several groups. Most tests used a combination of ambiguous situations and false beliefs, as well as understanding, and used environmental information to carry on behavior that, to be performed, needed EF modulation. Some others measured facial emotional recognition, which seems to be a process correlated with EFs, and assessed body, face, and speech cues during social interactions. Finally, some tests were employed in the selected studies to evaluate social behaviors that imply the capability to take into consideration social information and decide the most appropriate behavior and/or engage in social problem solving. Indeed, these capabilities need verbal and nonverbal working memory and shifting ability [59]. Related to these tests, DERS [60] is a questionnaire to evaluate emotional dysregulation. Indeed, several items of this test focus on dysregulated behaviors that could give rise to consequences during social interaction. Interestingly, emotional dysregulation in ADHD seems to be connected to EFs [59].

Table 3 shows the results of social cognition processes related to EFs. The selected studies assessed a variety of processes: emotion recognition [12,34,43,50]; matching emotional faces [11]; making complex social judgments and inferring second-order false beliefs [33]; the mental–physical distinction [33]; understanding ambiguous situations [4,50,54]; problem solving and social skills behavior [48,55,56]; emotional regulation [50]; processing of emotional situations, recognition of emotional faces, and social interactions [51]; emotional perception [34]; social problem solving [35]; and gestures codification [49].

Overall, the majority of the studies found that ADHD individuals showed some impairment in social cognition processes that need EFs compared to control subjects; however, some studies did not find any difference. Specifically, the studies that used matching emotional faces [11], emotional recognition and emotional perception tests [34], social problem solving [35], and ambiguous emotional faces and social decision making [54] did not find any difference between groups. Interestingly, the studies that did not find any difference tested only adults. Conversely, the studies that found some significant differences across groups tested adults and children. This discrepancy in the results might suggest that children with ADHD could show higher difficulties in regulating EFs, while adults could learn compensatory processes that may help them to regulate EFs and adapt to some complex social contexts.

## 4. Discussion

This systematic review aimed to synthesize recent findings on social cognition in ADHD to determine the specific processes that are altered in this population, leading to social relationship problems.

Overall, most studies assessing EFs and language showed significant differences in specific social cognitive processes between individuals with ADHD and the controls. Specifically, we found that ADHD individuals perform worse than the controls in the following language domains: pragmatics, facial expression recognition, use of context, social interest, inappropriate initiation, sarcasm/paradoxical sarcasm, and reading between the lines. We also found significant differences across EF processes and, in particular, in emotion recognition, social control, social problem solving, understanding false belief, metarepresentation, social judgment, and faux pas. Moreover, the studies showed that, in general, ADHD individuals tend to experience more emotional distress in social contexts compared to non-ADHD individuals. Conversely, regarding ToM, most studies found no differences between groups. Importantly, we also found that differences were more pronounced in children than adults, who may use compensatory processes that might help them regulate and adapt to the complexity of social contexts.

### 4.1. Language

Overall, the selected studies show some differences between ADHD and non-ADHD children in processes related to language. For instance, Geurts and Embrechts [29] examined verbal and nonverbal communication abilities, such as prosody, emotional expression, and content, in three groups of children with ADHD, ASD, and TD aged between 7 and 14 years. Interestingly, they found that the ASD and ADHD groups exhibited similar challenges in various language areas compared to the TD group. Specifically, both ASD and ADHD showed comparable difficulties in coherence, inappropriate initiation, and interest, although the results were not definitive. ASD showed the poorest performance in the use of context, nonverbal communication, and social relations, while ADHD displayed poorer performance in these areas compared to TD.

Other crucial aspects of language that were investigated in the selected studies are pragmatics and prosody. Pragmatics is defined as the ability to use speech appropriately, including initiating and concluding conversations, comprehending the general context of a conversation to predict parts of it, and maintaining coherence while telling a story. It was found that pragmatics is weaker in children with ADHD [29] and adolescents (aged 12–17) compared to healthy controls [50] but less severe in comparison to ASD. The other ability, prosody, allows us to interpret the intended meaning behind words, such as understanding humor, which is crucial for social information processing. It has been shown that prosody is another dimension of communication in which children with ADHD manifest problems [11,12]. Kis et al. [11] showed that children with ADHD have poor performance in emotional prosody and empathy tests. In particular, in the sub-test “not emotional prosody”, the authors showed no significant difference between groups, suggesting that there could be a correlation between the use of language and emotional context. Sahin et al. [48], who tested ADHD, ASD, and TD children to study language development and the understanding of unclear situations, found that ADHD and ASD individuals have difficulties in all studied domains.

Regarding paralanguage abilities assessed using DANVA, Demopoulos et al. [47] observed significant differences between ADHD and non-ADHD children in this test, while Cadesky et al. [43] did not. Two hypotheses could explain this difference: First, they examined different groups, as Cadesky et al. [43] studied ADHD versus CPs, while Demopoulos et al. [47] compared ADHD and ASD. This distinction might be crucial as Demopoulos et al. [47] did not consider language ability as an exclusion criterion due to communication challenges being a vital aspect of ASD. Conversely, Cadesky et al. [43] focused more on IQ differences.

Finally, only one study assessed language processes in adults and found significant differences in prosody across groups [11].

### 4.2. Theory of Mind

The reviewed studies show some differences across children and adults in processes related to ToM. In children, two studies underscored challenges faced by individuals with ADHD regarding certain facets of empathy. For instance, Sahin et al. [48] found that ADHD children performed poorer than TD in conceptualizing other’s mental states. Conversely, one study found that children and adolescents with ADHD can understand ToM concepts but struggle in social contexts [33]. Specifically, when exposed to basic concepts, they somewhat grasp contextual needs, desires, and emotional responses but not as well as individuals with TD. However, when faced with humor, hidden intentions, or behaviors that do not align with others’ desires, they exhibit poor performance and understanding of the situation [33].

In studies conducted on adults, the results show that ADHD children who exhibit ToM problems do not have these difficulties during adulthood [34,36,51,54,55]. Thus, suggesting that these challenges tend to improve as ADHD children grow into adults [11], possibly due to adaptation and learning of more complex ToM aspects or delayed skill development compared to non-ADHD individuals, as suggested by [61,62]. ADHD children may develop ToM abilities later than neurotypical children, similar to the development of cortical thinning. In social cognition situations, individuals with ADHD may learn other strategies to compensate for some difficulties in adulthood, similar to how they compensate in other situations, such as memory [63].

Regarding neural correlates, ToM is associated with specific brain areas linked to empathy, language, and EFs. Regarding the tests used in the selected studies to measure ToM, we can find evidence that they recruit different neural networks. For instance, in tests like the RMET, which require participants to see images of eyes and decide what emotion is expressed, brain areas, such as the left inferior frontal gyrus [64,65], left posterior superior temporal sulcus [65,66,67], and medial prefrontal cortex [67] are involved apart from the amygdala [65,66]. In other types of tasks, such as the social context film clips used by Friedman et al. [51], participants needed to understand the context before determining the emotion experienced by the character, which implies EF capabilities like focusing on multiple targets and considering implicit information. In Happè’s Strange Stories, used in Parke et al. [12], studies suggest that the posterior superior temporal sulcus, dorsomedial prefrontal cortex, and inferior frontal gyrus are involved. According to the functional parcellation model [68], the left inferior frontal gyrus is associated with phonological processes [69], the left posterior superior temporal sulcus is involved in conceptual matching [70,71], and the medial prefrontal cortex plays a role in memory, learning, and working memory [72,73], which are all part of EF capabilities. Studies suggest a reduced size of the amygdala [74,75,76] and abnormalities in its activation [77], as well as altered functioning in the other areas in populations with ADHD [78]. Therefore, it is possible to speculate that the impaired performance in RMET and other ToM tests like Happè’s Strange Stories could be due to the altered functioning of the amygdala, left posterior superior temporal sulcus, medial prefrontal cortex, and left inferior frontal gyrus.

### 4.3. Executive Functions

It has been suggested that EFs may play a key role in the functioning of individuals with ADHD. Barkley and Poillion [79] identified EF abilities that are altered in ADHD, such as response inhibition, working memory, shifting (the ability to move back and forth between tasks), planning, and executive behavior for future goals. A consequence of EF dysfunction is poor adaptation in social contexts, where impulsivity, hyperactivity, and attention deficit could lead to difficulties during interactions with others [15,16]. Considering social cognition aspects that involve EFs, we found that children with ADHD evidence difficulties in a variety of processes, such as gestural codification and nonverbal communication [43,49], emotion recognition and emotion regulation [48,51,52], and understanding ambiguous situations and sarcasm [4,46]. These difficulties seem less pronounced during adolescence than adulthood, during which they seem less intense or, in some cases, absent. Indeed, while all studies that considered children found significant differences, only three out of seven studies conducted in adults found that EF processes related to social cognition were impaired in individuals with ADHD compared to the controls [51,55,57].

Regarding the studies that did not find significant differences across all the processes (language, ToM, and EFs), we can consider two possible explanations. First, it is possible that tested patients were under pharmacological treatment (an aspect not considered in the exclusion criteria), making the performance influenced by drugs. In Thoma et al. [35], adult ADHD patients did not show a significant difference compared to the controls in both social cognition and EFs, but 16 out of 19 patients with ADHD used drugs, with 12 using MPH, 1 a combination of MPH and olanzapine, and 3 using bupropion. In Gonzalez-Gadea et al. [54], adult patients with ADHD were taking MPH (54.5%), atomoxetine (9.1%), and benzodiazepines (18.8%), while the rest of the patients with ADHD (36.3%), along with all individuals with Asperger Syndrome and the controls, were not following any pharmacotherapy. In Ilzarbe et al. [53], adult patients with ADHD used psychostimulant medications and selective serotonin reuptake inhibitors (SSRIs). Withdrawing 24 h before testing sessions and during neuroimaging data acquisition, the authors found some differences in neuronal correlates. In Mehren et al. [36], the authors took into consideration the possibility of pharmacological influences, conducting two separate statistical analyses, excluding four adult ADHD patients who were under pharmacological treatment and also excluding four adult controls for matching purposes. These results also showed no significant difference in ToM tests between individuals with ADHD and control groups. In the other studies, we observed a certain degree of variability. Specifically, in five studies, participants suspended medications 24 h before testing [4,12,43,52,56], while in twelve studies, participants did not take any medication [11,29,33,34,44,45,47,48,49,50,51,55]. Finally, in the Ludlow et al. study [46], all ADHD participants were taking prescribed medication for ADHD. Overall, given the high heterogeneity in the exclusion criteria, it is possible that these null results on social cognition might have been, to some degree, influenced by the effect of the medication.

Another explanation could be linked to the developmental stage. Indeed, there is evidence suggesting that in ADHD development, there are differences in cortical thinning [80], indicating that the pruning phenomenon occurs around two years later than in neurotypical development. However, in a study by Hauser et al. [81], in which an ADHD adolescent group and a control group performed a probabilistic learning task under fMRI, the authors found minor activation of the medial prefrontal cortex during the task and they did not find any significant difference in performance. These results might suggest that individuals with ADHD may use other brain areas to achieve a similar result.

## 5. Limitations

This study has some limitations. First, we did not include any gray literature. Gray literature is important because it can influence the review synthesis and publication bias [82]. Hence, there may be existing studies that are or are not documented in the gray literature and these could include results that influence the review’s conclusions. The second limitation relates to the search engine. The literature search was conducted using three search engines, PsychInfo, Pubmed, and Google Scholar, and no other databases were searched. Therefore, additional relevant studies might have been missed. Third, we included all systematic reviews with available full texts written in English and excluded articles published in preprint databases due to lack of peer review. Fourth, the data considered were often different regarding questionnaires submitted to participants, experimental design, and statistical processes, so a meta-analysis was not performed due to the high level of methodological variability. An additional limitation was that our focus was on EFs, ToM, and language aspects of social cognition, overlooking other cognitive abilities that could impact social cognition in individuals with ADHD. Finally, we did not take into account that individuals in this specific population are often vulnerable to traumatic events [83] and attachment issues [84] that could affect certain aspects of social cognition tied to interpersonal biases, as seen in clinical practice in personality disorders [85,86]. Therefore, further research is necessary to understand social cognition biases in the ADHD population, for instance, by taking into account these relevant variables.

## 6. Conclusions

From the 22 studies reviewed, we found that across language, ToM, and EF processes related to social cognition, most of the studies show some significant differences between ADHD and non-ADHD individuals, especially in language and EFs compared to ToM. Moreover, these differences were more pronounced in children compared to adults. This pattern of results across ages may depend on the larger body of research performed on children compared to adults and on the compensatory processes learned during development that might enhance performance during adulthood [61].

These results align with the perspective that difficulties in social cognition are not solely attributable to the core manifestations of ADHD, such as hyperactivity, impulsivity, and inattention. Instead, they may also arise from additional mechanisms related to social cognition as a whole. This suggests a need to investigate the functioning of brain regions implicated in social cognition within this population. It is plausible that certain cognitive processes develop at a different pace compared to individuals without ADHD, leading to atypical social cognition functioning. Alternatively, individuals with ADHD may rely on different neural pathways to support social adaptability and knowledge, resulting in general difficulties with social cognitive abilities. Future research should explore these avenues to further evaluate social cognition in individuals with ADHD.

Our results may have important implications in the clinical field. Indeed, during the therapeutic process, it could be fundamental to provide evidence of specific social cognition ability that is impaired or atypical in ADHD patients to work on and improve their quality of life regarding social interaction and social adaptation.

## Figures and Tables

**Table 1:** Description of studies that investigated the relationship between social cognition and language.

Study	Groups (N)	Gender [M/F]	Age [Children/Adolescents]	Measures	Function and Process	Main Results	Impairment
Cadesky et al. (2000)	ADHD (86); ADHD + CP (63) CP (24); HC (27)	[5:1] [7:1] [6:1]	9.0 9.3 9.3 9.3	DANVA	Language: nonverbal communication	No differences among groups	No
Kim and Kaiser (2000)	ADHD (11) TD (11)	[10/1] [3/7]	6–8; 6–8	PPVT-R TOPL pragmatic protocol	Language: pragmatic skills	ADHD performed worst in sentence imitation and word articulation. Differences in speech language quotient and pragmatic behaviors. In addition, they produced more inappropriate pragmatic behaviors.	Yes
Geurts, and Embrechts, (2008)	ADHD (29); ASD (29); TD (29)	[28/1] [28/1] [28/1]	10.19; 10.10; 10.13	CCC-2 (language skills in social communication)	Language: verbal and nonverbal communication	ADHD performed worse than TD but better than ASD in the use of context, nonverbal communication, social relations, and general pragmatic score. ADHD performed worse than TD but similar to ASD in coherence, inappropriate initiation, interest, and general communication score.	Yes
Demopoulos et al. (2013)	ADHD (436); ASD (137)	[293/143]; [123/14]	10.58; 10.39	DANVA (affect recognition); CASL (pragmatic language)	Language: nonverbal communication and pragmatic skills	ADHD performed poorer compared to normative data and better than ASD on all tests.	Yes
Staikova et al. (2013)	ADHD (28); TD (35)	[23/25]; [24/11]	8.62; 9.08	CCC-2 (language skills in social communication); TOPL (pragmatic); NAP (oral discourse); CASL (pragmatic abilities); TOLP-2 (pragmatic); CELF-4 (verbal language); SSIS (social behavior in children)	Language: verbal and nonverbal communication	ADHD showed general poorer pragmatic skills. ADHD showed poorer performance in discourse management and narrative discourse. Discourse management fully mediated the relation between ADHD and social skills.	Yes
Ludlow et al. (2017)	ADHD (22); TD (22)	[15/7]; [15/7]	12.9; 11.8	TASIT (test sincerity, sarcasm, and paradoxical sarcasm recognition)	Language: sincerity, sarcasm, and paradoxical sarcasm recognition	ADHD showed poorer performance in identification of exchanges. Sincere exchanges were recognized significantly more often than simple sarcasm and paradoxical sarcasm; simple sarcasm was identified more than paradoxical sarcasm.	Yes
Sahin et al. (2018)	ADHD (24); ASD (26); SLD (24); TD (24)	[18/6]; [23/3]; [16/8]; [20/4]	9; 9.5; 9; 10	Hinting task (reading between the lines)	Language: reading between the lines	ADHD, ASD, and SLD performed poorer than control group in faux pas, TFBL, and hinting task. No differences among these clinical groups.	Yes
Parke et al. (2021)	ADHD (25); TD (25)	[19/6]; [15/10]	10.57; 10.07	CCC-2 (pragmatic language)	Language: pragmatic skills	ADHD children exhibited deficits compared to TD in pragmatic skills.	Yes
Çiray et al. (2022)	ADHD (70); TD (64)	[51/19]; [46/18]	14.41; 14.77	CCC-2 (verbal and nonverbal communication); SRS-2 (social interaction skills, language skills, and restricted interest)	Language: verbal and nonverbal communication and interaction skills	ADHD performed worse than TD in speech, syntax, semantics, coherence, inappropriate initiation, stereotype language, nonverbal communication, and social relations.	Yes
Kis et al. (2017)	ADHD (28); TD (29)	[18/10]; [14/15]	33.8; 36.5	TAB	Language and emotional prosody	ADHD showed a poorer performance in emotional prosody discrimination, and name emotional prosody.	Yes

Description of studies included that investigated social cognition considering aspects of language cognitive function. Abbreviations: ADHD = Attention Deficit Hyperactivity Disorder; TD = Typically Developed; CP = Conduct Problem; HC = Healthy Control; ASD = Autism Spectrum Disorder; SLD = Specific Learning Disorder; PPVT-R = Peabody Picture Vocabulary Test Revised; TOLP = Test of Pragmatic Language; DANVA = Diagnostic Analysis of Nonverbal Accuracy; CCC-2 = Children’s Communication Checklist-2; NAP = Narrative Assessment Profile; CASL = Comprehensive Assessment of Spoken Language; CELF-4 = Clinical Evaluation of Language Fundamentals; SSIS = Social Skills Improvement System; TAB = Tübinger Affect Battery; SRS-2 = Social Responsiveness Scale Second Edition; TASIT = Awareness of Social Inference Test.

**Table 2:** Description of studies that investigated the relationship between social cognition and ToM.

Study	Groups (N)	Gender [M/F]	Age [Children/Adolescents]	Measures	Function and Process	Main Results	Impairment
Hutchins et al. (2016)	ADHD (29); TD (49); ASD (67)	[29/0]; [49/0]; [67/0]	9.02; 8.82; 9.77	ToMTB (explicit ToM competence)	ToM	No difference between ADHD and TD. ASD performed worse.	No
Sahin et al. (2018)	ADHD (24); ASD (26); SLD (24); TD (24)	[18/6]; [23/3]; [16/8]; [20/4]	9; 9.5; 9; 10	REMT	ToM	ADHD, ASD, and SLD performed poorer than control group REMT. No difference among clinical groups.	Yes
Çiray et al. (2022)	ADHD (70); TD (64)	[51/19]; [46/18]	14.41; 14.77	RMET (ToM)	ToM	ADHD performed worse than TD in RMET.	Yes
Parke et al. (2021)	ADHD (25); TD (25)	[19/6]; [15/10]	10.57; 10.07	RMET (Affective ToM)	ToM	ADHD children exhibited deficits compared to TD in affect recognition.	Yes
**Study**	**Groups (N)**	**Gender [M/F]**	**Adults**	**Measures**	**Function and Process**	**Main Results**	**Impairment**
Friedman et al. (2003)	ADHD (31); TD (32)	[19/12]; [16/16]	37.7; 36.7	Social context film clips (emotional understanding)	ToM	No significant differences.	No
Gonzalez-Gadea et al. (2013)	ADHD (22); AS (23); TD (21)	[14/8]; [15/8]; [11/10]	35.2; 33.0; 38.2	RMET	ToM: emotional comprehension	No significant differences.	No
Kis et al. (2017)	ADHD (28); TD (29)	[18/10]; [14/15]	33.8; 36.5	CBS (empathy)	ToM: empathy	ADHD showed poorer performance in empathy—CBS.	Yes
Pitzianti et al. (2017)	ADHD (10); TD (10)	[14/9]; [11/9]	33.1; 33.0	RMET (infer the affective mental states of others)	ToM	A trend (but not significant) in RMET in which ADHD performed worse.	
Abdel-Hamid et al. (2019)	ADHD (30); TD (30)	[15/15]; [15/15]	34.50; 35.83	MASC	ToM: emotional comprehension	No significant differences.	No
Ilzarbe et al. (2020)	ADHD (21); ASD (19); ADHD + ASD; TD (25)	[21/0]; [19/0]; [18/0]; [25/0]	23.1; 23.0; 23.1; 23.4	Frith–Happé animated triangle fMRI task (ToM)	ToM: understanding the intention of others	No difference in performance among the groups.	No
Mehren et al. (2021)	ADHD (26); TD (26)	[21/5]; [21/5]	31.0; 28.5	MASC (ecologically cognitive and affective ToM); brain morphometry measures (dispositional empathy).	ToM: emotional comprehension	No significant results.	No
Tatar and Cansız (2022)	ADHD (40); TD (40)	[22/18]; [22/18]	21.72; 21.75	RMET	ToM: facial expression, comprehension, and social emotions	ADHD individuals performed significantly worse than TD in ToM.	Yes

Description of studies included that investigated social cognition considering aspects of ToM cognitive function. Abbreviations: ADHD = Attention Deficit Hyperactivity Disorder; TD = Typically Developed; ASD = Autism Spectrum Disorder; SLD = Specific Learning Disorder; AS = Asperger’s Syndrome; ToM = Theory of Mind; CBS = Cambridge Behavior Scale; RMET = Reading Mind in the Eyes Test; TOMTB = Theory of Mind Task Battery; MASC = Movie for the Assessment of Social Cognition; FC = Functional Connectivity; rTPJ/STS = Temporoparietal Junction/Superior Temporal Sulcus.

**Table 3:** Description of studies that investigated the relationship between social cognition and EF.

Study	Groups (N)	Gender [M/F]	Age [Children/Adolescents]	Measures	Function and Process	Main Results	Impairment
Cadesky et al. (2000)	ADHD (86); ADHD + CP (63); CP (24); HC (27)	[5:1]; [7:1]; [6:1]; [1.5:1]	9.0; 9.3; 9.3; 9.3	Battery: DANVA (adult and child facial expressions)	EF: emotion recognition	ADHD and CP performed worst in adult and child facial expression.	Yes
Demopoulos et al. (2013)	ADHD (436); ASD (137)	[293/143]; [123/14]	10.58; 10.39	TOPS (social problem solving); BASC-2 (parent rating of social skills)	EFs: problem solving and social skills behavior	ADHD performed poorer compared to normative data and better than ASD in facial affect, vocal affect, pragmatic judgment, and problem solving.	Yes
Hutchins et al. (2016)	ADHD (29); TD (49); ASD (67)	[29/0]; [49/0]; [67/0]	9.02; 8.82; 9.77	ToMI (applied ToM)	EFs: making complex social judgments, inferring second-order false beliefs, social referencing, reading basic emotions, basic metarepresentation, seeing leads to knowing, and the mental–physical distinction	ADHD and ASD performed poorer compared to TD in applied ToM. TD performed better than ADHD, and ADHD performed better than ASDS in early subscale score (social referencing and reading basic emotions). Specifically, DHD performed better than ASD in basic subscale scores: (basic metarepresentation, seeing leads to knowing and the mental–physical distinction. In particular, TD performed better than ADHD in advanced subscale scores (making complex social judgments and inferring second-order false beliefs).	Yes
Sahin et al. (2018)	ADHD (24); ASD (26); SLD (24); TD (24)	[18/6]; [23/3]; [16/8]; [20/4]	9; 9.5; 9; 10	FPR	EF: ambiguous situation	ADHD, ASD, and SLD performed poorer than control group in faux pas. No difference among clinical groups.	Yes
Maoz et al. (2019)	ADHD (24); TD (36)	[16;8]; [19/7]	10.29; 9.37	FPR (recognize social faux pas); IRI (cognitive and affective empathy)	EF: understanding ambiguous situations	Significant difference between ADHD and TD in recognizing social faux pas. There was a significant difference in the two cognitive empathy subscales (IRI perspective taking and IRI fantasy). Another difference was found in one affective empathy subscale (IRI empathic concern).	Yes
Hilton et al. (2020)	high ADHD (20); Low ADHD (20)	[11/9]; [11/9]	10.10; 9.55	PONS	EF: gesture codification	High ADHD performed significantly worse than low ADHD in social encoding test.	Yes
Parke et al. (2021)	ADHD (25); TD (25)	[19/6]; [15/10]	10.57; 10.07	IRI; NEPSY-II (social cognitive	EFs: emotional recognition	ADHD children exhibited deficits compared to TD in NEPSY affect recognition and cognitive empathy.	Yes
Parke et al. (2021)	ADHD (25); TD (25)	[19/6]; [15/10]	10.57; 10.07	Happe’s Strange Stories (cognitive ToM)	ambiguous situations, sarcasm, and misunderstanding	ADHD children exhibited deficits compared to TD in cognitive ToM.	Yes
Çiray et al. (2022)	ADHD (70); TD (64)	[51/19]; [46/18]	14.41; 14.77	Faces Test (emotional recognition); DERS (emotional regulation); FPR (understanding of ambiguous situations);	EFs: ambiguous situations, emotional recognition, and emotional regulation	ADHD performed worse than TD in SRS, Faces Test, comprehension test, emotional dysregulation, emotional dysregulation impulsivity, and non-acceptance.	Yes
**Study**	**Groups (N)**	**Gender [M/F]**	**Age [Adults]**	**Measures**	**Function and Process**	**Main Results**	**Impairment**
Friedman et al. (2003)	ADHD (31); TD (32)	[19/12]; [16/16]	37.7; 36.7	LIWC (affect and social processing); Benton Test of Facial Recognition (facial perception); SSI (social skills)	EFs: processing of emotional situations, recognition of emotional faces, m and social interactions	ADHD perceived themselves as less competent in social control and social expressivity than TD.	Yes
Gonzalez-Gadea et al. (2013)	ADHD (22); AS (23); TD (21)	[14/8]; [15/8]; [11/10]	35.2; 33.0; 38.2	PFT; IGT	EFs: ambiguous emotional faces and social decision making	No significant differences.	No
Kis et al. (2017)	ADHD (28); TD (29)	[18/10]; [14/15]	33.8; 36.5	TAB	EF: matching emotional faces	No significant differences.	No
Pitzianti et al. (2017)	ADHD (10); TD (10)	[14/9]; [11/9]	33.1; 33.0	Dual valence task (discrimination of positive or negative affect face). Presence of N170 (cortical marker implicated in facial processing)	EFs: emotional recognition and emotional perception	No significant results.	No
Abdel-Hamid et al. (2019)	ADHD (30); TD (30)	[15/15]; [15/15]	34.50; 35.83	Cambridge Behavior Scale	EF: social behavior	ADHD showed lower average score in empathy; EF seems to moderate empathy performance.	Yes
Thoma et al. (2020)	ADHD (19); TD (20)	[9/10]; [10/10]	36.2; 36.7	IRI (dispositional empathy); Mentalistic Interpretation Task (understanding others’ mental state during difficult interpersonal situation); social problem Resolution Task (finding adequate solution for difficult everyday interpersonal solution); social problem-solving fluency task (ability to generate high quality solution in awkward social situation).	EF: social problem solving	In IRI, ADHD showed higher scores in personal distress subscale.	No
Ugurpala et al., 2023	ADHD (30); TD (30); SAD (30); SAD-ADHD (30)	[15/15]; [13/17]; [15/15]; [17/13]	23.5; 23.3; 22; 22.7	DEToMI	EFs: first-order false belief, second-order false belief, irony, metaphor, and faux pas recognition tasks	In first-degree false belief, ADHD showed lower performance than TD and SAD, whereas SAD + ADHD patients showed lower performance than TD. In irony, SAD patients showed better performance than ADHD patients. In empathic comprehension, ADHD showed lower performance than the other groups, whereas SAD and SAD + ADHD groups showed a similar performance compared to the HC group.	Yes

Description of studies included that investigated social cognition considering aspects of EF cognitive function. Abbreviations: ADHD = Attention Deficit Hyperactivity Disorder; CP = Conduct Problem; HC = Healthy Control; TD = Typically Developed; ASD = Autism Spectrum Disorder; AS = Asperger Syndrome; SLD = Specific Learning Disorder; SAD = Social Anxiety Disorder; DANVA = Diagnostic Analysis of Nonverbal Accuracy; TAB = Tübinger Affect Battery; ToMI = Theory of Mind Inventory; DEToMI= Dokuz Eylül Theory of Mind Index; FPR = Faux Pas Recognition Task; IRI = interpersonal reactivity index; TOPS = Test of Problem Solving; BASC = Social Skills Scale of the Behavior Assessment Scale for Children Second Edition; DERS = Difficulties in Emotion Reactivity Scale; SSI = Social Skills Inventory; LIWC = Linguistic Inquiry and Word Count Dictionary; PONS = Profile of Nonverbal Sensitivity; IGT = Iowa Gambling Task; SRS = Social Reciprocity Scale Second Edition.

## Data Availability

Not applicable.

## Supplementary Materials

The following supporting information can be downloaded at https://www.mdpi.com/article/10.3390/brainsci14111117/s1, Figure S1. PRISMA Checklist; Table S1. Risk of Bias.
